# *R*-Limonene Enhances Differentiation and 2-Deoxy-D-Glucose Uptake in 3T3-L1 Preadipocytes by Activating the Akt Signaling Pathway

**DOI:** 10.1155/2018/4573254

**Published:** 2018-09-03

**Authors:** Ilavenil Soundharrajan, Da Hye Kim, Srigopalram Srisesharam, Palaniselvem Kuppusamy, Ki Choon Choi

**Affiliations:** ^1^Grassland and Forage Division, National Institute of Animal Science, Rural Development Administration, Cheonan 31000, Republic of Korea; ^2^Laboratory of Animal Physiology, Graduate School of Agricultural Science, Tohoku University, Aoba, Sendai 980-8577, Japan

## Abstract

Adipocyte is an important place for lipid storage. Defects in lipid storage in adipocytes can lead to lipodystrophy and lipid accumulation in muscle, liver, and other organs. It is the condition of mixed dyslipidemia which may favor the development of insulin resistance via lipotoxic mechanisms. Our objective of the study was to investigate the potential role of* R*-limonene (LM) on differentiation, lipid storage, and 2-deoxy-D-glucose (2DG) uptake in 3T3-L1 preadipocytes. Genes and proteins associated with differentiation, lipid accumulation, 2DG uptake and its signaling pathways in the adipocytes were analyzed using qPCR and western blot methods. LM treatment increased differentiation, lipid accumulation, and the expression of adipogenic and lipogenic markers such as C/EBP-*α*, C/EBP-*β*, PPAR*γ*, SREBP-1, RXR, FAS, and adiponectin. However, the LM concentration at 10*μ*M decreased (p < 0.05) adipogenesis and lipogenesis via regulating key transcriptional factors. LM treatment increased activation of Akt by increasing its phosphorylation, but p44/42 activation was not altered. MK-2206, an Akt specific inhibitor, reduced the activation of Akt phosphorylation whereas LM treatment aborted the MK-2206 mediated inhibition of Akt activation. LM enhanced glucose uptake in differentiated adipocytes. Overall data suggested that LM treatment favored lipid storage and glucose uptake in adipocytes via activation of key transcriptional factors through activation of Akt phosphorylation in 3T3-L1 adipocytes.

## 1. Introduction

Differentiation of adipocytes is an essential process for the lipid storage. Inhibiting adipocyte differentiation alone is not an appropriate way to prevent obesity because adipocytes provide a safe place for lipid accumulation. Defects in adipogenesis can lead to stimulating lipodystrophy. In this condition, lipids are accumulated in muscle, liver, and other organs which develop the insulin resistance [1, 2]. Animal and human experiments have proved that type 2 diabetes mellitus is closely associated with adipogenesis and its factors [3].

Adipogenesis is a complex process that is regulated by a cascade of transcription factor and other regulatory proteins. Numerous positive and negative factors have been involved in adipocyte differentiation [4–7]. Among these, C/EBP-*β* (CCAAT/enhancer-binding protein-*β*) and C/EBP-*δ* act together and induce the expression of PPAR-*γ* 2 (peroxisome proliferator-activated receptor) and C/EBP-*α* (CCAAT/enhancer-binding protein-*α*). These factors are known to regulate genes and proteins associated with adipogenesis and lipogenesis [8].

The Akt kinases play a critical role in adipogenesis and glucose transport [9]. Lack of or inhibition of Akt activation in fibroblast displays an inability to differentiate preadipocyte into adipocytes. In addition, activation of Akt can promote the differentiation of preadipocytes into mature adipocytes even in the absence of other factors involved in adipogenesis. Activation of Akt can induce the peroxisome proliferator-activated receptor expression which is the key transcriptional factor in the adipocyte differentiation and lipid accumulation [10–14].

**Figure 1:**
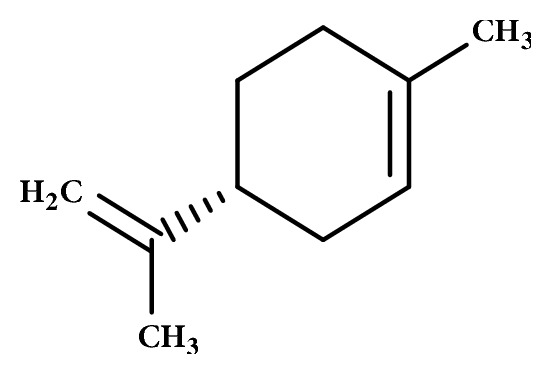
The molecular structure of* R*-limonene (LM).

Monoterpenes are the major constituents of the plant kingdom. They are the primary contributor to the organoleptic properties associated with different herbs, spices, citrus fruits, most flowers, spearmint oil, pine oil, and fruits [15]. Monoterpenes are a class of terpenes that consist of two isoprene units and it has many biological functions including the broad spectrum of antimicrobial activities, allelopathic, herbivore deterring, pollinator attracting properties, antioxidant, antiphlogistic, antitumor, antiviral, and antinociceptive properties. In addition, they can stimulate glucose uptake in C2C12 cells [16–19]. Among monoterpenes, limonene (Figure 1) and *α*-pinene exhibit potent antitumor properties [20, 21]; in addition, limonene has been shown to be helpful in relieving heartburn and gastroesophageal reflux disorder; it supports normal peristalsis [22].

Limonene can enhance glucose uptake in 3T3-L1 adipocytes via glucose transporters [23]. It possesses antidiabetic effects by preventing dyslipidemia [24]. It also prevents insulin resistance [25], LM enhanced mitochondrial biogenesis, and elevated protein levels of hormonal sensitive lipase (HSL), perilipin (PLIN), AMP-activated protein kinase (pAMPK), Phospho acetyl CoA carboxylase (pACC), Acy coenzyme A oxidase (ACO), cytochrome c oxidase subunit 4 (COX4), carnitine palmitoyltransferase 1 (CPT1), and cytochrome C (CYT-C) at the concentration of 25 and 50*μ*M of limonene, which are involved in lipolysis and lipid catabolism via activation of *β*-adrenergic receptor-3 (*β*3-AR) and extracellular signal-regulated kinases (ERK) signaling pathway [26]. Dietary intake of monoterpenes by the human is relatively high. LM with lemons-like odor has been used as a fragrance additive in candy, ice cream, orange juice, and chewing gum. Human consumes both synthetic and natural limonene at a range of 0.27 milligrams per kilogram of body weight per one day. Consumption of monoterpenes could provide significant health beneficial effects especially anti-breast cancer and anti-colorectal carcinoma effects. It strengthens the role of dietary monoterpenes in human life [22]. An early study indicated that* D-*limonene exhibited antiobesity activity in adipocytes and high-fat diet induced obesity at the concentration of 50*μ*M and 0.5% limonene, respectively [27].

Lipodystrophic patients have a defect in triglyceride storage in adipose tissues which causes lipid accumulation elsewhere in the body developing severe insulin resistance. Increases of fat capacity storage in adipose with low net mobilization lead to the expansion of fat mass and it may also be considered as the best way to store the lipids in harmless compartment [2]. In this aspect, we planned to investigate LM effects on positive regulation of differentiation, lipid accumulation, and glucose uptake in 3T3-L1 adipocytes. In addition, the molecular mechanisms involved in the effect of LM on differentiation and glucose uptake were explored in this study.

## 2. Materials and Methods

### 2.1. Cell Culture and Chemicals

The 3T3-L1 preadipocytes cell line was obtained from the American Type Culture Collection [Rockville, MD, USA]. Dulbecco modified Eagle medium [DMEM] and fetal bovine serum (FBS) were procured from Gibco-BRL [Gaithersburg, MD, USA]. Kits for mRNA extraction, cDNA synthesis, and qPCR were purchased from Bio-Rad [Hercules, CA, USA].* R*-limonene (Sigma Aldrich, #183164) and rosiglitazone were obtained from Sigma Aldrich (St. Louis, MO, USA). MK-2206 was obtained from Selleckchem (Houston, TX, USA). Monoclonal antibodies (PPAR*γ*, RXR, C/EBP-*α*, SREBP-1, aP2, FAS, adiponectin, ACC, tAKT, pAKT (Serine 473), p44/42, pp44/42 (Thr 202/ Tyr204), AMPK-*α*, pAMPK-*α* (Thr172), and GAPDH) used in the study were obtained from Cell Signaling Technology (Danvers, MA, USA).

### 2.2. Cytotoxicity of Limonene (LM)

Ez-cytox assay kit (iTSBiO, Korea) was used to determine the cytotoxic effects of LM. Briefly, 3T3-L1 preadipocytes (ATCC, USA) were seeded into 96-well cell culture plates at the density of 1 × 10^4^ cells/well and incubated at 37°C with 5% CO_2_ for 24 hours. These cells were then treated with different concentrations of cyclic terpene limonene (LM) and incubated for 1 day, 2 days, and 10 days; every 48 h fresh media was replaced with different concentration of LM until the end of experimental periods. Ten microliters of water-soluble tetrazolium (WST) reagent was added to each well and incubated at 37°C with 5% CO_2_ for 1-2 hours. Absorbance (color intensity) of each well was measured at a wavelength of 450 nm using a Spectra Count microplate plate reader [28].

### 2.3. Differentiation Induction

3T3-L1 preadipocytes were seeded into 6- and 12-well cell culture plates at a density of 3 × 10^4^ and 1.5 × 10^4^ cells/well, respectively, and incubated at 37°C with 5% CO_2_. Every 48 h, the culture medium was replenished with fresh medium. When cells reached 100%, further incubation was performed for another two days. Growth medium was then replaced with a differentiation induction medium (DMI) (0.5 mM 3-isobutyl-1-methylxanthine, 1 *μ*M dexamethasone, and 1 *μ*g/mL insulin) containing LM, agonist, and antagonist [28].

### 2.4. Quantification of Lipid Using Oil Red O Staining

Differentiated 3T3-L1 adipocytes in 6-well plates were fixed with 2 mL of 10% formalin for 1 h and then rinsed with 40% isopropanol. After rinsing, 3 mL of Oil Red O staining solution was added to each well. Plates were incubated at room temperature for 15 min and washed three times with distilled water. Stained cells were photographed with an inverted microscope [CKX41, Olympus Corporation, Tokyo Japan]. Additionally, Oil Red O stain was eluted with 100% isopropanol and measured at 490 nm [28].

### 2.5. Glucose Uptake Assay

Eight-day differentiated cells were incubated with serum-free media for 12 h. Then, serum- starved cells were treated with LM (5*μ*M), insulin (10*μ*M), and MK-2206, an antagonist for Akt (8nM) individually for 2 h at 37°C with 5% CO_2_ followed by addition of 10 mM 2DG and incubated for another 20 min. Glucose uptake was then performed using glucose uptake assay kit according to the manufacturer's protocol (glucose uptake colorimetric assay kit, Abcam, UK)

### 2.6. Quantification of Gene Expression Using Quantitative RT-PCR

Total RNA was extracted from experimental cells using RNeasy lipid mini kit (Qiagen, MD, USA) and quantified by Spectramax i3 (Molecular devices, CA, USA). Total RNA (500 ng) was then reverse transcribed using iScript cDNA synthesis kit (Hercules, CA, USA). Gene transcripts in the experimental samples were quantified with SYBR Green-based qPCR using specific primers for C/EBP-*α* (F-gcaggaggaagatacaggaag, R- acagactcaaatccccaaca); C/EBP-*β* (F-gtttcgggacttgatgcaatc, R- aacaaccccgcaggaaca); PPAR *γ*2 (F-gtgctccagaagatgacagac, R-ggtgggactttcctgctaa); Adiponectin (F-ccgttctcttcacctacgac, R- tccccatccccatacac); FAS (F-cccagcccataagagttaca, R-atcgggaagtcagcacaa); SREBP-1 (F-gaagtggtggagacgcttac, R-tatcctcaaaggctggactg); *β*-actin (F-ctctccctcacgccatc, R- atgtcacgcacgatttcc) on a CFX 96 Real Time PCR detection system (Hercules, CA, USA). All gene expressions were normalized against housekeeping gene *β*-actin [28].

### 2.7. Protein Extraction and Immunoblotting

Proteins were extracted from experimental adipocytes with RIPA lysis buffer containing protease and phosphatase inhibitors cocktail (Roche, Switzerland and Sigma Aldrich, USA). Protein quantification was performed with the Pierce BCA protein assay kit (Thermo Fisher Scientific, MA, USA). Immunoblotting was performed with Cell Signaling Technology monoclonal antibodies [29]. The intensity of immunoreacted bands was quantified with ImageJ software version 1.49 (32 bit), (Wayne Rasband, National Institute of Health, USA).

### 2.8. Statistical Analysis

Experimental data were subjected to one-way ANOVA with post hoc test and multivariate comparisons with Duncan test using the statistical package of social science [SPSS-Version 16.0, SPSS, Inc., USA]. Statistical significance was considered when* p* value was less than 0.05.

## 3. Results

### 3.1. Cytotoxic Effects of LM on 3T3-L1 Preadipocytes

**Figure 2:**
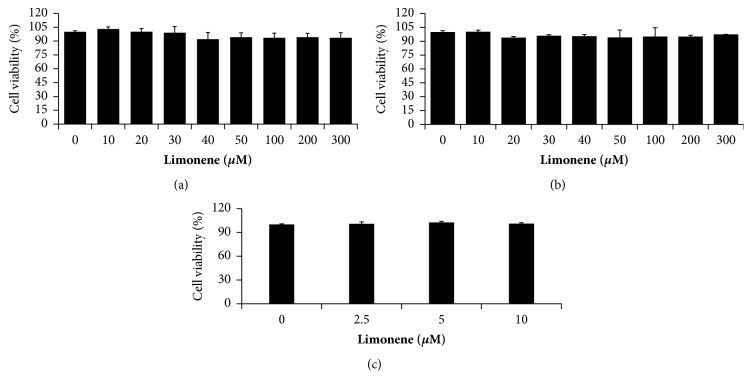
Cytotoxic effect of LM on 3T3-L1 preadipocytes. (a) LM effect on 3T3-L1 preadipocytes viability after 24 h treatment; (b) LM effect on 3T3-L1 preadipocytes viability after 48 h treatment; (c) LM effect on 3T3-L1 preadipocytes viability after 10 days of treatment.

Preadipocytes were treated with different concentrations (10*μ*M to 300 *μ*M**)** of LM and incubated for 1 day, 2 days, and 10 days. LM treatment failed to affect the cell viability significantly at a concentration of 10 *μ*M or less. Furthermore, the increase of LM concentration slightly reduced cell viability compared to the control. However, there was no significant difference in cell viability between control and LM treatment at any concentration tested (Figure 2).

### 3.2. Effects of LM on 3T3-L1 Preadipocyte Differentiation

**Figure 3:**
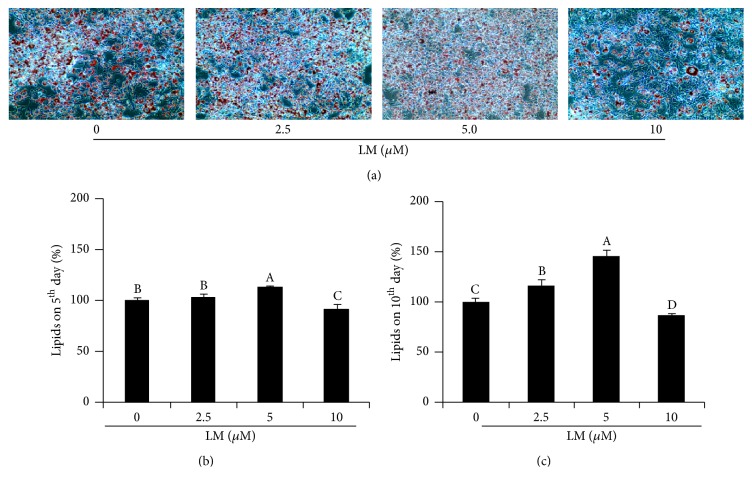
Impact of LM on differentiation and lipid accumulation in 3T3-L1 adipocytes. (a) Oil Red O stained lipid droplets in control and LM treated adipocytes on day 10. (b) Percentage of lipid accumulation in experimental adipocytes on day 5. (c) Percentage of lipid accumulation in experimental adipocytes on day 10. Experiments were performed in six replicates and repeated three times with the same results. Bars display mean ± SEM and statistical analysis was performed by one-way ANOVA. Different letters, A, B, C, and D, within a column indicate a statistically significant difference between the groups (*p* < 0.05).

Based on microscopic observation, adipocytes treated with differentiation media started to increase the accumulation of lipid droplets in adipocytes (Figure 3). Such lipid accumulation and differentiation were accelerated (*p* < 0.05) after the addition of LM at 2.5 *μ*M and 5 *μ*M compared to those in control adipocytes. Lipid accumulation in maturing adipocytes after treatment with LM at 10 *μ*M was reduced (*p* < 0.05) compared to that in control adipocytes. After extracting Oil Red O dye from experimental adipocytes, higher absorbance values in adipocytes treated with LM at 2.5 and 5 *μ*M but lower absorbance value in adipocytes treated with LM at 10 *μ*M compared to control adipocytes were observed, indicating that the LM could regulate the lipid accumulation in adipocytes in a dose dependent-manner. Maximum lipid accumulation was noted in adipocytes on the 5th and 10th day after treatment with 5 *μ*M of LM without any alteration in cell morphology. Therefore, we used 5 *μ*M of LM for further experiment.

### 3.3. Effect of LM on mRNA Expression in 3T3-L1 Adipocytes

**Figure 4:**
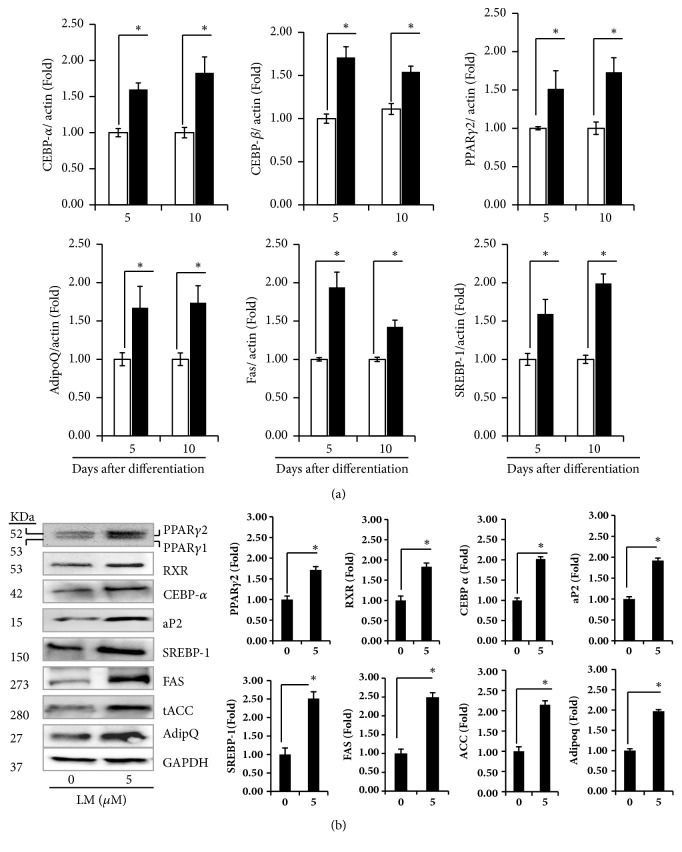
(a) Impact of LM on adipogenic and lipogenic mRNA expression in 3T3-L1 adipocytes. (b) Effects of LM on adipogenic and lipogenic proteins on day 10. Confluent 3T3-L1 adipocytes were differentiated with DMI in the presence/absence of LM for 10 days. Experiments were performed in triplicate and repeated three times with the same results. Bars display mean ± SEM and statistical analysis was performed by one-way ANOVA. *p* < 0.05 indicates a statistically significant difference between the control and LM treated adipocytes.

We next investigated how LM regulated the expression of PPAR*γ* and C/EBPs in adipocytes. Expression levels of C/EBP-*α*, C/EBP-*β*, and PPAR*γ* were significantly (*p* < 0.05) upregulated in adipocytes treated with LM at 5 *μ*M compared to those in control adipocytes. The expression levels of adiponectin, FAS, and SREBP-1 mRNAs were also significantly increased by LM treatment compared to those in control adipocytes (Figure 4(a)). LM treatment at a concentration of 10 *μ*M significantly (*p* < 0.05) downregulated PPAR*γ*, C/EBP-*α*, C/EBP-*β*, SREBP-1, FAS, and adiponectin mRNA expression levels in differentiated adipocytes compared to control (data not shown).

### 3.4. Effect of LM on Adipogenic and Lipogenic Proteins Expression in 3T3-L1 Adipocytes

Expression levels of protein expressions related to adipocyte differentiation in adipocytes on the 10th day after treatment with LM were analyzed. Treatment with LM at 5 *μ*M significantly (p < 0.05) increased protein levels of PPAR*γ*, RXR, C/EBP-*α*, SREBP-1, and their downstream targets such as aP2, FAS, ACC, and adiponectin, compared to control adipocytes (Figure 4(b)).

### 3.5. Comparative Effects of LM and Rosiglitazone (RGZ) on Differentiation and PPAR*γ* Expression

**Figure 5:**
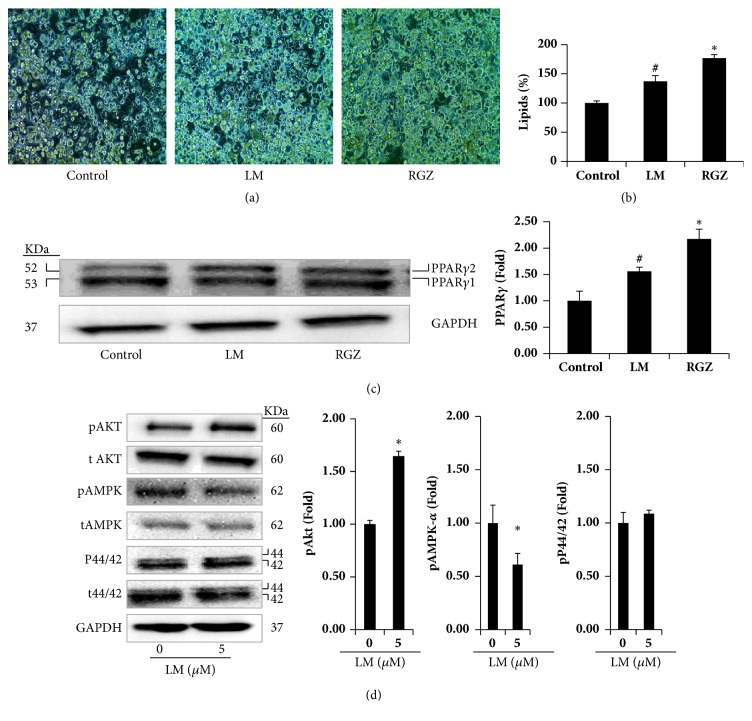
(a)–(d) A comparative study between* R-*limonene (LM) and rosiglitazone (RGZ) on lipid accumulation and PPAR-*γ* expression in adipocytes. Adipocytes differentiated with RGZ (0.1*μ*M) and LM (5*μ*M) separately in DMI. (a) Microscopic visualization of differentiated adipocytes on day 10; (b) percentage of lipids in the experimental adipocytes on day 10; (c) PPAR-*γ* protein expression in the experimental adipocytes on day 10. Experiments were performed in triplicate and repeated three times with the same results. Bars display mean ± SEM and statistical analysis was performed by one-way ANOVA. *∗*p<0.05 indicates a statistically significant difference compared to control and LM treatments; #p<0.05 indicates a statistically significant difference compared to control adipocytes. (d) Impact of LM on signaling pathways associated with an adipocyte differentiation. Adipocytes were differentiated with differentiation induction medium (DMI) in the presence/absence of LM for 10 days. Experiments were performed in triplicate and repeated three times with the same results. Bars display mean ± SEM and statistical analysis was performed by one-way ANOVA. *∗*p<0.05 indicates a statistically significant difference compared to control adipocytes.

We also compared the effects of LM with those of PPAR-*γ* agonist (RGZ) in adipocytes. Confluent preadipocytes were treated with RGZ or LM (0.1 *μ*M or 5 *μ*M, respectively) for 48 h. Results showed that RGZ treatment significantly increased lipid accumulation and PPAR-*γ* protein expression. Similarly, LM treatment significantly increased lipid accumulation and PPAR-*γ* protein expression compared to control adipocytes (Figures 5(a)–5(c)).

### 3.6. Effects of LM on Signaling Pathways

Next, signaling pathways involved in the regulation of adipocyte differentiation and lipid accumulation in control and LM treated adipocytes were investigated. LM treatment induced activation of Akt by increasing its phosphorylation at serine 473 but decreased AMPK-*α* phosphorylation at Thr172 in adipocytes on the 10th day compared to the control adipocytes. However, p44/42 pathway remained the same in the control and experimental adipocytes (Figure 5(d)).

### 3.7. A Competitive Study between LM and MK-2206 on Akt Phosphorylation

**Figure 6:**
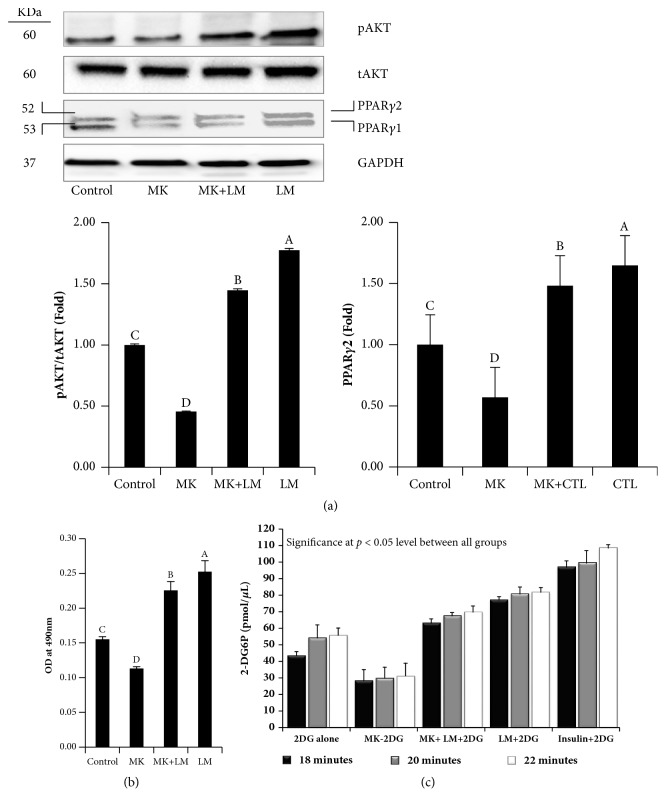
(a)-(b) A competitive study between LM and MK-2206 on Akt phosphorylation at serine 473 during the differentiation of 3T3-L1 preadipocytes. Adipocytes were differentiated in DMI with LM (5*μ*M), or MK-2206 (8nM), or LM + MK-2206 individually. LM alone or cotreatment of LM with MK-2206 increased PPAR-*γ* and Akt activation. MK-2206 alone treated adipocytes exhibited downregulation of PPAR-*γ* and Akt activation. (a) Western blot analysis of experimental proteins. (b) An optical density of extracted Oil Red O stains from experimental adipocytes. Experiments were performed in triplicate and repeated three times with the same results. Bars display mean ± SEM and statistical analysis was performed by one-way ANOVA. Different letters, A, B, and C, within a column indicate a statistically significant difference (*p* < 0.05). (c).** Effects of LM on 2DG glucose uptakes in 3T3-L1 adipocytes**. Differentiated adipocytes in differentiation induction medium (DMI) were exposed to LM (5*μ*M), Insulin (10*μ*M), MK-2206 (8nM), and MK+LM separately for 2 h and glucose uptake was then analyzed in 3T3-L1 adipocytes using a glucose uptake assay kit with kinetic mode (Abcam, USA). Experiments were performed in six replicates and repeated three times with the same results. Bars display mean ± SEM and statistical analysis was performed by one-way ANOVA. Significance was at* p* < 0.05 level between the groups.

MK-2206, an antagonist of Akt, was used in this study to further evaluate the role of Akt involved in the effect of LM on adipocyte differentiation. Adipocytes treated with MK-2206 for 48 h inhibited lipid accumulation and downregulated PPAR-*γ* expression compared to the control adipocytes. Cotreatment with both LM and MK-2206 (Akt inhibitor) significantly (*P *< 0.05) increased lipid accumulation and upregulated PPAR-*γ* expression in differentiated adipocytes, indicating that LM treatment could abolish MK-2206 mediated downregulation of PPAR-*γ* and optical density of Oil Red O stain. These data confirmed that LM treatment could increase differentiation and lipid accumulation via Akt activation by increasing its phosphorylation at serine 473 (Figures 6(a) and 6(b)).

### 3.8. LM on Glucose Uptake in Differentiated 3T3-L1 Adipocytes

Differentiated adipocytes were then treated with LM, insulin, MK-2206, and MK-2206+LM separately and incubated for 2 h in a CO_2_ incubator after serum starvation. Insulin treatment increased 2DG uptake in 3T3-L1. Treatment with MK-2206, an antagonist for Akt, reduced glucose uptakes. LM treatment significantly enhanced 2DG uptake in 3T3-L1 adipocytes. MK-2206 + LM treatment also increased glucose uptakes as compared to control adipocytes. Overall data suggest that the LM could stimulate 2DG uptake in differentiated adipocytes through the Akt signaling pathway in differentiated adipocytes (Figure 6(c)).

## 4. Discussion

Adipogenesis is a sequential process accompanied by the dramatic increase in the expression of adipocyte genes [7]. The dysfunctional adipose tissue is characterized by reduced adipogenesis, increased cellular senescence, and inflammation. Such dysfunction impairs lipid storage and dysregulates production of adipokines and cytokines, leading to metabolic disorder, adipose tissue inflammation, insulin resistance, and type-2 diabetes [30]. An adequate adipogenesis is essential to sequester lipids in adipose tissues to prevent ectopic fat deposition and insulin resistance development. Increases in lipid storage capacity are believed to play a vital role in metabolic regulation [31]. In the present study, LM treatment induced differentiation and lipid accumulation in adipocytes at 5th and 10th day after treatment. However, cells treated with LM at the concentration higher than 10 *μ*M inhibited lipid accumulation with slight morphology changes compared to control.

Furthermore, we analyzed effects of LM on expression levels of C/EBP-*β*, PPAR*γ*, and C/EBP-*α* known to be essential factors that work sequentially and cooperatively to induce differentiation and lipid accumulation in adipocytes [7]. In the present study, LM induced C/EBP-*β*, C/EBP-*α*, PPAR- *γ*2, and RXR expression. Crosstalks among PPAR- *γ*, RXR, and C/EBP*α* at common regulatory sites are important in the regulation of genes involved in adipocyte differentiation, for lipogenic function requires PPAR- *γ*/RXR heterodimers while the vital function is mediated by PPAR- *γ*/RXR *α* or PPAR- *γ* / RXR*ɣ* heterodimers [32]. C/EBP-*β* is mainly involved in adipocyte differentiation. It activates key transcriptional factors such as PPAR *γ* and C/EBP-*α* [33]. Upregulation of these genes was consistent with lipid accumulation results based on Oil Red O staining.

Adiponectin is exclusively secreted in fully differentiated adipocytes. It regulates many metabolic processes via enhancing insulin sensitivity in muscle and liver or by activating fatty acid oxidation in different tissues [34]. In the present study, LM treatment increased mRNA of adiponectin and their protein expression levels in differentiated adipocytes compared to control, suggesting that LM might promote the glucose uptake by cells.

PPAR*γ* and C/EBP-*α* can regulate their downstream targets such as aP2, FAS, LPL, leptin, and adipoQ. These downstream targets can trigger the synthesis of fatty acids and triglycerides synthesis [5]. Adipocytes can dramatically induce lipogenesis after differentiation induction, leading to insulin sensitivity. FAS, aP2, and ACC are known to be increased 10- to 100-fold during differentiation [35]. In the present study, LM treatment increased FAS, ACC, and adiponectin levels in differentiated adipocyte as compared to the control, indicating that LM may trigger insulin sensitivity via activating adipocyte differentiation and fatty acid metabolism by regulating key transcriptional factors and their downstream targets. Adipocyte binding protein (aP2) is a key mediator involved in the transport of fatty acids and their metabolism regulation. It is regulated by PPAR*γ* during differentiation and lipid accumulation [36]. LM significantly upregulated aP2 expression in differentiated adipocytes as compared to the control. SREBPs can enhance genes associated with cholesterol and fatty acid biosynthesis and uptake [37]. Furthermore, SREBP-1c induces PPAR*γ* expression in adipocytes. In collaboration with C/EBP-*α*, SREBP-1c can activate adipocyte markers [38]. Furthermore, activation of AMPK directly phosphorylates precursor of SREBP-1c at ser 372 residue which controls proteolytic maturation and translocation of matured SREBP-1c into the nucleus which leads to reducing the expression of ACC and FAS [39–41]. The present study is indicating that LM treatment could increase mature SREBP-1c expression via AMPK-*α* downregulation and increased ACC and FAS expression levels which are the key enzymes involved in the lipogenesis.

The insulin signaling pathway plays a crucial role in preadipocyte differentiation [29]. Akt is particularly important in adipocyte differentiation and insulin metabolic functions [42]. Many researchers have reported that Akt regulates PPAR*γ* and adipocyte differentiation [12, 43]. In the present study, insulin, DEX, and IBMX mixture increased Akt phosphorylation at ser 473. Furthermore, activation of Akt by increasing its phosphorylation was accelerated by LM treatment. These data suggest that activation of Akt can activate genes associated with adipocyte differentiation. It is known that Akt phosphorylation can promote adipocyte differentiation via upregulating PPAR *γ* [44]. In addition, cotreatment with Akt inhibitor and LM significantly increased lipid accumulation and differentiation, indicating that LM treatment could abort inhibition of Akt phosphorylation induced by Akt inhibitor and increase phosphorylation of Akt. Taken together, these results suggest that LM might accelerate adipogenesis and lipogenesis via Akt signaling pathway in adipocytes. In the meantime, the Erk1/2 pathway remained the same in control and LM treated cells.

Finally, we examined the effect of LM on glucose uptake properties of cells because LM upregulated Akt phosphorylation at serine 473 and adiponectin level in 3T3-L1 adipocytes. It is known that insulin signaling pathway is activated under nutrient availability. GLUT-4 translocation is required for insulin-dependent PI3K/AKT activation [45]. Akt kinase, a Ser/ Thr kinase, is activated by insulin and certain growth factors. Several reports have shown that Akt function is downstream of the PI3 kinase. Akt kinase critically contributes to glucose uptake and metabolism through PI3K kinase in the insulin signaling pathway [14]. In this study, adipocyte treated with LM or insulin increased glucose uptake in 3T3-L1 adipocytes. However, MK-2206, a specific inhibitor for Akt activation, caused a reduction in glucose uptake. These results confirmed that LM could enhance glucose uptake in adipocytes via activating Akt signaling pathway.

## 5. Conclusions

In summary, the present study showed that limonene (LM) could induce differentiation and glucose uptake in 3T3-L1 preadipocytes. LM regulated adipogenesis and lipogenesis via induction of key transcriptional factors such as PPAR*γ*, C/EBP-*α*, and C/EBP-*β* as well as their downstream targets by activation of Akt signaling pathway. Our overall data suggest that limonene is a promising compound found in the natural products that can favor lipid storage in adipocytes and glucose uptake via the Akt signaling pathway.

## Data Availability

The data used to support the findings of this study are available from the corresponding author upon request.

## References

[1] Samuel V. T., Petersen K. F., Shulman G. I. (2010). Lipid-induced insulin resistance: unravelling the mechanism. *The Lancet*.

[2] Virtue S., Vidal-Puig A. (2010). Adipose tissue expandability, lipotoxicity and the Metabolic Syndrome—an allostatic perspective. *Biochimica et Biophysica Acta (BBA) - Molecular and Cell Biology of Lipids*.

[3] Dubois S. G., Heilbronn L. K., Smith S. R., Albu J. B., Kelley D. E., Ravussin E. (2006). Decreased expression of adipogenic genes in obese subjects with type 2 diabetes. *Obesity*.

[4] Lefterova M. I., Lazar M. A. (2009). New developments in adipogenesis. *Trends in Endocrinology & Metabolism*.

[5] Cristancho A. G., Lazar M. A. (2011). Forming functional fat: a growing understanding of adipocyte differentiation. *Nature Reviews Molecular Cell Biology*.

[6] Christy R. J., Kaestner K. H., Geiman D. E., Daniel Lane M. (1991). CCAAT/enhancer binding protein gene promoter: Binding of nuclear factors during differentiation of 3T3-L1 preadipocytes. *Proceedings of the National Acadamy of Sciences of the United States of America*.

[7] Gregoire F. M., Smas C. M., Sul H. S. (1998). Understanding adipocyte differentiation. *Physiological Reviews*.

[8] White U. A., Stephens J. M. (2010). Transcriptional factors that promote formation of white adipose tissue. *Molecular and Cellular Endocrinology*.

[9] Kajno E., McGraw T. E., Gonzalez E. (2015). Development of a new model system to dissect isoform specific Akt signalling in adipocytes. *Biochemical Journal*.

[10] Peng X.-D., Xu P.-Z., Chen M.-L. (2003). Dwarfism, impaired skin development, skeletal muscle atrophy, delayed bone development, and impeded adipogenesis in mice lacking Akt1 and Akt2. *Genes & Development*.

[11] Baudry A., Yang Z. Z., Hemmings B. A. (2006). PKBalpha is required for adipose differentiation of mouse embryonic fibroblasts. *Journal of Cell Science*.

[12] Yun S.-J., Kim E.-K., Tucker D. F., Kim C. D., Birnbaum M. J., Bae S. S. (2008). Isoform-specific regulation of adipocyte differentiation by Akt/protein kinase B*α*. *Biochemical and Biophysical Research Communications*.

[13] Xu J., Liao K. (2004). Protein kinase B/AKT 1 plays a pivotal role in insulin-like growth factor-1 receptor signaling induced 3T3-L1 adipocyte differentiation. *The Journal of Biological Chemistry*.

[14] Kohn A. D., Summers S. A., Birnbaum M. J., Roth R. A. (1996). Expression of a constitutively active Akt Ser/Thr kinase in 3T3-L1 adipocytes stimulates glucose uptake and glucose transporter 4 translocation. *The Journal of Biological Chemistry*.

[15] Crowell P. L., Gould M. N. (1994). Chemoprevention and therapy of cancer by d-limonene. *Critical Reviews in Oncogenesis*.

[16] Bakkali F., Averbeck S., Averbeck D., Idaomar M. (2008). Biological effects of essential oils—a review. *Food and Chemical Toxicology*.

[17] Choi H.-S., Song H. S., Ukeda H., Sawamura M. (2000). Radical-scavenging activities of citrus essential oils and their components: detection using 1,1-diphenyl-2-picrylhydrazyl. *Journal of Agricultural and Food Chemistry*.

[18] Kamatou G. P. P., Vermaak I., Viljoen A. M., Lawrence B. M. (2013). Menthol: a simple monoterpene with remarkable biological properties. *Phytochemistry*.

[19] Alkhateeb H., Bonen A. (2010). Thujone, a component of medicinal herbs, rescues palmitate-induced insulin resistance in skeletal muscle. *American Journal of Physiology-Regulatory, Integrative and Comparative Physiology*.

[20] Rabi T., Bishayee A. (2009). Terpenoids and breast cancer chemoprevention. *Breast Cancer Research and Treatment*.

[21] Bhattacharjee B., Chatterjee J. (2013). Identification of proapoptopic, anti-inflammatory, anti- proliferative, anti-invasive and anti-angiogenic targets of essential oils in cardamom by dual reverse virtual screening and binding pose analysis. *Asian Pacific Journal of Cancer Prevention*.

[22] D-Limonene S. (2007). D-Limonene: safety and clinical applications. *Alternative Medicine Review*.

[23] Tan X. C., Chua K. H., Ram M. R., Kuppusamy U. R. (2016). Monoterpenes: novel insights into their biological effects and roles on glucose uptake and lipid metabolism in 3T3-L1 adipocytes. *Food Chemistry*.

[24] Murali R., Karthikeyan A., Saravanan R. (2013). Protective effects of D-limonene on lipid peroxidation and antioxidant enzymes in streptozotocin-induced diabetic rats. *Basic & Clinical Pharmacology & Toxicology*.

[25] Santiago J. V. A., Jayachitra J., Shenbagam M., Nalini N. (2012). Dietary d-limonene alleviates insulin resistance and oxidative stress-induced liver injury in high-fat diet and L-NAME-treated rats. *European Journal of Nutrition*.

[26] Lone J., Yun J. W. (2016). Monoterpene limonene induces brown fat-like phenotype in 3T3-L1 white adipocytes. *Life Sciences*.

[27] Jing L., Zhang Y., Fan S. (2013). Preventive and ameliorating effects of citrus D-limonene on dyslipidemia and hyperglycemia in mice with high-fat diet-induced obesity. *European Journal of Pharmacology*.

[28] Choi K., Roh S.-G., Hong Y.-H. (2003). The role of ghrelin and growth hormone secretagogues receptor on rat adipogenesis. *Endocrinology*.

[29] Ilavenil S., Da Kim H., Srigopalram S. (2016). Potential application of p-coumaric acid on differentiation of C2C12 skeletal muscle and 3T3-L1 preadipocytes-an in vitro and in silico approach. *Molecules*.

[30] Addison W. N., Fu M. M., Yang H. X. (2014). Direct transcriptional repression of Zfp423 by Zfp521 mediates a bone morphogenic protein-dependent osteoblast versus adipocyte lineage commitment switch. *Molecular and Cellular Biology*.

[31] Kim S. M., Lun M., Wang M. (2014). Loss of white adipose hyperplastic potential is associated with enhanced susceptibility to insulin resistance. *Cell Metabolism*.

[32] Metzger D., Imai T., Jiang M. (2005). Functional role of RXRs and PPAR*γ* in mature adipocytes. *Prostaglandins, Leukotrienes and Essential Fatty Acids*.

[33] Tanaka T., Yoshida N., Kishimoto T., Akira S. (1997). Defective adipocyte differentiation in mice lacking the C/EBP*β* and/or C/EBP*δ* gene. *EMBO Journal*.

[34] Naowaboot J., Chung C. H., Pannangpetch P. (2012). Mulberry leaf extract increases adiponectin in murine 3T3-L1 adipocytes. *Nutrition Research*.

[35] Paulauskis J. D., Sul H. S. (1988). Cloning and expression of mouse fatty acid synthase and other specific mRNAs. Developmental and hormonal regulation in 3T3-L1 cells. *The Journal of Biological Chemistry*.

[36] Sun L., Nicholson A. C., Hajjar D. P., Gotto A. M., Han J. (2003). Adipogenic differentiating agents regulate expression of fatty acid binding protein and CD36 in the J744 macrophage cell line. *Journal of Lipid Research*.

[37] Shimomura I., Bashmakov Y., Ikemoto S., Horton J. D., Brown M. S., Goldstein J. L. (1999). Insulin selectively increases SREBP-1C mRNA in the livers of rats with streptozotocin-induced diabetes. *Proceedings of the National Acadamy of Sciences of the United States of America*.

[38] Fajas L., Schoonjans K., Gelman L. (1999). Regulation of peroxisome proliferator-activated receptor *γ* expression by adipocyte differentiation and determination factor 1/sterol regulatory element binding protein 1: Implications for adipocyte differentiation and metabolism. *Molecular and Cellular Biology*.

[39] Li Y., Xu S., Mihaylova M. M. (2011). AMPK phosphorylates and inhibits SREBP activity to attenuate hepatic steatosis and atherosclerosis in diet-induced insulin-resistant mice. *Cell Metabolism*.

[40] Magaña M. M., Lin S. S., Dooley K. A., Osborne T. F. (1997). Sterol regulation of acetyl coenzyme A carboxylase promoter requires two interdependent binding sites for sterol regulatory element binding proteins. *Journal of Lipid Research*.

[41] Ha J.-H., Jang J., Chung S.-I., Yoon Y. (2016). AMPK and SREBP-1c mediate the anti-adipogenic effect of hydroxyisovalerylshikonin. *International Journal of Molecular Medicine*.

[42] Shearin A. L., Monks B. R., Seale P., Birnbaum M. J. (2016). Lack of AKT in adipocytes causes severe lipodystrophy. *Molecular Metabolism*.

[43] Zhang H. H., Huang J., Düvel K. (2009). Insulin stimulates adipogenesis through the Akt-TSC2-mTORC1 pathway. *PLoS ONE*.

[44] Yoshiga D., Sato N., Torisu T. (2007). Adaptor protein SH2-B linking receptor-tyrosine kinase and Akt promotes adipocyte differentiation by regulating peroxisome proliferator-activated receptor *γ* messenger ribonucleic acid levels. *Molecular Endocrinology*.

[45] Towler M. C., Hardie D. G. (2007). AMP-activated protein kinase in metabolic control and insulin signaling. *Circulation Research*.

